# Editorial: MiniResource papers from *Molecules and Cells*

**DOI:** 10.1016/j.mocell.2026.100344

**Published:** 2026-03-10

**Authors:** Yejin Hwang, Seung-Jae V. Lee

**Affiliations:** Department of Biological Sciences, Korea Advanced Institute of Science and Technology, 291 Daehak-ro, Yuseong-gu, Daejeon, South Korea, 34141

## Abstract

In this editorial, we provide an integrative overview of MiniResource papers published in *Molecules and Cells* in 2024 and 2025, which establish reliable methodological practices across various fields in molecular and cellular biology. By emphasizing quantitative validation, analytical rigor, and reproducible experimental design, this collection serves as a practical guide for biological discovery.

Molecular and cellular biology advances fast when researchers turn complex measurements into reliable routines. Scientists often confront the same bottlenecks as experiments fail to reproduce across laboratories and pipelines vary when tools evolve. *Molecules and Cells* turned to this idea, publishing a collection of MiniResources in recent years. These methodological guides collectively strengthen experimental rigor in research fields of genome editing, transcriptomics, proteomics, imaging, model organisms, physiology, and computational biology. This curated collection published in 2024 and 2025 provides a practical resource for experimental researchers by providing guidelines for what to measure and how to validate.

## Genetic perturbation and molecular reconstruction

CRISPR-Cas9 genome editing in mammalian cells is a powerful strategy for genetic manipulation. A concise protocol for generating single-cell-derived knockout clones details steps for single guide RNA design, vector design, and next-generation sequencing validation (Hong et al., 2024a). An overview of the principles of gene cloning offers experimental considerations for restriction, ligation strategy selection, and increasing transformation efficiency (Hong et al., 2025). At the organismal level, a guide for stereotaxic delivery of recombinant viral vectors into the mouse brain introduces a structured practice for spatially regulating genetic modulation *in vivo* (Han et al., 2025).

## Functional interpretation of transcriptomics data

The expansion of high-throughput sequencing has enabled transcriptome-level analysis. A practical guide to bulk RNA sequencing provides an accessible resource for quality control, differential expression, and enrichment analysis (Lee et al., 2024a). Methodological reliability for measuring RNA levels in various samples is maintained through a protocol for reverse transcription-quantitative PCR (Bong et al., 2024). For those extending resolution to cellular heterogeneity, a resource for single-cell RNA sequencing addresses ambient RNA correction, batch effect mitigation, and clustering strategies (Kim et al., 2024a). The bottleneck for many transcriptomic studies is interpretation through analysis pipelines that fail when quality control, sample size, or modeling choices weaken statistical power. The expression quantitative trait locus guide details genotype processing, variant filtering, and statistical modeling schemes for users (Ko et al., 2024). To refine biological interpretation, GOREA (Gene Ontology-R-enrichment analysis) introduces hierarchical clustering and enrichment ranking to improve Gene Ontology biological process summarization (Lee et al., 2025). THRAISE (transcriptome-wide high-throughput RNA-seq analysis integrated system), an automated and reproducible web platform, further supports standardization of transcriptomic processes that consolidate RNA-seq processing pipelines (Heo and Um, 2025). OASIS portable offers secure offline survival analysis infrastructure (Han et al., 2024), extending computational accessibility for OASIS and OASIS2 (Han et al., 2016; Yang et al., 2011). A curated overview of essential R packages provides researchers with structured tools for genomic, transcriptomic, proteomic, and text-mining analyses (Chua et al., 2024).

## Biochemical and cellular analysis tools

Quantitative biochemical characterization is addressed through determining dissociation constants (K_d_) (Lee et al., 2024b) and assessing mitochondrial membrane potential, stress-response, and intracellular calcium (Park and Kang, 2025). A TurboID analysis guide for plants provides a roadmap for proximity labeling coupled with mass spectrometry for interactome mapping (Park and Kim, 2025). READRetro introduces a web-based platform for plant retrosynthetic pathway prediction, integrating metabolic engineering and computational chemistry (Kwak et al., 2025). Plasma membrane protein detection methodologies further support accurate surface protein analysis via labeling and biotinylation strategies (Roh et al., 2025). In multiparametric guides to ferroptosis and cellular senescence, markers including lipid peroxidation, iron metabolism (Nghiem et al., 2025), morphological changes, and senescence-associated secretory phenotypes (SASPs) (Kang et al., 2024) allow accurate detection. Mechanistic understanding requires reliable protein-level interrogation. A concise guide to western blot assays outlines antibody validation and detection chemistry (Ha et al., 2025), whereas immunostaining protocols address antigen retrieval, signal amplification, and optimization (Park et al., 2025a).

## Experimental approaches to cell biology

Quantitative cellular phenotyping depends on accurate imaging and multiparametric analysis. Flow cytometry can accurately quantify heterogeneous cell populations only when panel design, compensation, and gating are rigorously executed. A detailed guide to flow cytometry supplies stepwise guidelines for such rigor (Song and Lee, 2024). Complementary methodologies for immune cell isolation from lymphoid and nonlymphoid tissues standardize cell preparation prior to downstream functional analysis (Park and Lee, 2025), while structured protocols for murine T cell isolation and activation strengthen immunological experimentation (Lim et al., 2025). Single-molecule fluorescence resonance energy transfer (FRET)-based approaches extend dynamic protein interaction analysis at high spatial resolution (Kim et al., 2024b), and optimization of FRET imaging in plant protoplast systems enables precise assessment of protein-protein interactions in plant biology (Choi et al., 2025). An illustration of how investigators can visualize endogenous enhancer-promoter contacts within nuclei answers how chromatin architecture supports transcription using chromatin labeling (Park and Cho, 2024). To complement measurement strategies, SimpleViz offers unbiased functional enrichment interpretation and visualization of the data (Oh et al., 2025).

## Model organisms and in vivo systems

Cross-study comparisons of model organism data often vary when performing assays with different protocols. For *Caenorhabditis elegans*, practical guidance on strain acquisition streamlines experimental setup (Park et al., 2025), while survival assays and health parameter measurements help standardize lifespan and stress response analyses (Kwon and Lee, 2025, Hwang et al., 2025). Imaging and quantitative analysis instructions support reproducible phenotyping in *C. elegans* (Min et al., 2025). A standard for zebrafish-based experimentation provides structured embryotoxicity testing protocols that address breeding and morphological assessment (Hong et al., 2024b). Open-source software tools enhance zebrafish embryo behavioral analysis and quantitative phenotyping (Ranasinghe and Cha, 2025). Essential resources and best practices for laboratory mouse research consolidate strain selection, database utilization, and ethical considerations under the 3Rs: replacement, reduction, and refinement for humane experimental techniques (Haque et al., 2025).

This curated MiniResource collection from *Molecules and Cells* in 2024 and 2025 provides a methodological ground for biological discovery. By promoting standard procedures and documenting reliable practices, this collection provides the scientific community with an essential toolkit for high-impact research.

## Funding and Support

None.

## Author Contributions

Y.H. and S-J.V.L. wrote this article.

## Declaration of Generative AI and AI-Assisted Technologies in the Writing Process

During the preparation of this work, ChatGPT-5.4 (OpenAI) and Gemini 3 (Google) were used to improve the clarity of the language. The authors reviewed and revised the content after using these tools and take full responsibility for the content of the publication.

## Declaration of Competing Interests

The authors have no potential conflicts of interest to disclose.
